# Adherence to standard treatment guidelines among prescribers in primary healthcare facilities in the Dodoma region of Tanzania

**DOI:** 10.1186/s12913-021-06257-y

**Published:** 2021-03-24

**Authors:** Karin Wiedenmayer, Eva Ombaka, Baraka Kabudi, Robert Canavan, Sarah Rajkumar, Fiona Chilunda, Selemani Sungi, Manfred Stoermer

**Affiliations:** 1 Swiss Tropical and Public Health Institute, P.O. Box, CH-4002 Basel, Switzerland; 2 University of Basel, P.O. Box, CH-4003 Basel, Switzerland; 3 St. John’s University Tanzania, Dodoma, Tanzania; 4 Mission for Essential Medical Supplies, P.O. Box 1005, Arusha, Tanzania; 5 Health Promotion and System Strengthening project, Dodoma, Tanzania

**Keywords:** Standard treatment guidelines, Adherence, Prescribers, Primary healthcare facilities, Antibiotics prescription, Rational use of medicines, Tanzania

## Abstract

**Background:**

Tanzania’s primary healthcare system suffers from a scarcity of financial and human resources that impedes its effectiveness to deliver dependable and uniform quality healthcare. Adherence to standard treatment guidelines (STG) can help provide more consistent and correct diagnoses and treatments and limit the irrational use of medicines and the negative health consequences that can occur as a result. The purpose of this study was to investigate prescribers’ adherence of their diagnoses and respective treatments to national STG and to identify potential areas for planning interventions.

**Methods:**

A cross-sectional study on prescribers’ adherence to diagnosis and treatment, according to national STG, was conducted in 2012 in public primary healthcare facilities (HCF) in the Dodoma region of Tanzania. Information on 2886 patients was collected, prospectively and retrospectively, from 120 HCF across the Dodoma region using a structured questionnaire. Twenty-five broadly defined main illness groups were recorded and the nine most prevalent and relevant conditions were statistically analysed in detail.

**Results:**

Diagnoses and related treatments were recorded and analysed in 2872 cases. The nine most prevalent conditions were upper respiratory tract infections (25%), malaria (18%), diarrhoea (9.9%), pneumonia (6.1%), skin problems (5.8%), gastrointestinal diagnoses (5%), urinary tract infections (4%), worm infestations (3.6%) and eye problems (2.1%). Only 1.8% of all diagnoses were non-communicable diseases. The proportion of prescribers’ primary diagnoses that completely adhered to national STG was 599 (29.9%), those that partially adhered totalled 775 (38.7%), wrong medication was given in 621 cases (30.9%) and no diagnosis or medication was given in nine cases (0.5%). Sixty-one percent of all patients received an antibiotic regardless of the diagnoses. Complete adherence was highest when worms were diagnosed and lowest for diarrhoea. The proportion of cases that did not adhere to STG was highest with patients with skin problems and lowest for malaria.

**Conclusion:**

Prescribers’ general adherence to national STG in primary HCF in the public sector in Dodoma region is sub-optimal. The reasons are multifaceted and focused attention, directed at improving prescribing and pharmacotherapy, is required with a view of improving patient care and health outcomes.

**Supplementary Information:**

The online version contains supplementary material available at 10.1186/s12913-021-06257-y.

## Background

Considerable global efforts are required to achieve the commitments and targets of the United Nations Sustainable Development Goal (SDG) 3, to ‘Ensure healthy lives and promote well-being for all at all ages,’ [1]. Achieving and maintaining universal healthcare (UHC), in particular, is an increasing challenge in the face of a continuing global economic crisis precipitated by the Corona Virus pandemic and its effects on the global healthcare budget, maximum efficiency among limited resources is therefore required. The rational use of medicines is an essential strategy to achieve optimal outcomes in healthcare by minimizing the threats posed by inappropriate treatment such as antimicrobial resistance, excessive use of intravenous medication that can lead to blood-borne diseases, illnesses due to under-prescribing, wastage and sickness due to polypharmacy [2]. Responsible prescribing and the adherence to standard treatment guidelines (STG) therefore needs optimizing in order to reduce disease burden. This is especially true for low- and middle-income countries (LMICs) where the irrational use of medicines is more widespread than in higher-income settings [3–7].

Yet, LMICs are often ill equipped to effectively manage the challenges attributable to the irrational use of medicines and prescribers non-adherence to STG. The decision-making process of PHC workers can be facilitated, e.g., by offering clinical assessment assisting software, as seen in the Netherlands, one of Europe’s most conservative antibiotic prescribers [8]. Although, the same approach is not yet an option in countries with a less developed digital environment such as the United Republic of Tanzania (hereinafter Tanzania). Increased consultation times, technical challenges using the devices, lack of qualified staff and financial motivation are some of the reasons for low uptake of such devices [9]. Nevertheless, several small promising efficacy and effectiveness studies of electronic clinical diagnostic algorithm tools, which have the potential to improve diagnosis and treatment to further advance the rational use of medicines in low resource settings, have been carried out [10]. However, what functions well in one clinical situation and health system context is not necessarily effective in another. As a point of reference, STG therefore need to be current, evidence-based and adapted to the local context. In addition, they need to be disseminated in combination with training, supervision and feedback [6, 11–13].

The Tanzanian STG and National Essential Drugs List was first printed in 1991 with further editions printed in 1997, 2007, 2013 and 2017. There are a limited number of studies on prescribers’ adherence to national STG; however, prescribers’ low usage, ownership or knowledge of existing STG was recorded in 2012 with additional negative feedback regarding, among others, their complexity and the complex language used [14].

A later study in 2017, concerning the adherence to malaria STG among healthcare workers in Meatu, Tanzania, demonstrated slightly better prescriber awareness and access to STG. Nevertheless, usage remained low and fewer strictly adhered to them [15]. Despite the introduction of newer STG editions, the inappropriate and irrational use of medicines and prescribing habits persist. Irunde and colleagues revealed in 2017 that the overprescribing of antibiotics, among others, continued to be an issue [3]. In addition, adherence appeared to be lower in rural healthcare facilities (HCF) compared to that of urban HCF. Furthermore, public HCF appeared to be slightly more compliant than private ones. However, a promising way forward could be to expand antimicrobial stewardship (AMS) programmes to improve the utilization of antibiotics across Tanzania. They have been established in some LMICs with limited but promising results. Interventions such as clinician education, protocol development and continuous reviews of guideline compliance have, inter alia, led to an increase in prescriber compliance with STG and reductions in antibiotic prescribing [16].

The Tanzanian Government aims to strengthen the health system country-wide so it can progress towards its Development Vision 2025 and SDG 3, including UHC. In 2011, the Health Promotion and System Strengthening (HPSS) project was introduced in Tanzania to support the Tanzanian Government with this aim by applying a comprehensive approach to health system strengthening within the health financing, medicines, health promotion and technology management sectors [17–19]. This study was part of the initial phase of the HPSS project. The aim was to explore the adherence of PHC workers diagnoses and respective treatments to national STG with the intent to inform any future interventions. The main research questions to ascertain adherence were: i) do prescribers comply with good prescribing practices; ii) do prescribers comply with the national STG; iii) are there differences in adherence to STG for different target groups; and iv) are there differences in adherence to STG for different diseases?

## Methods

### Study area

A cross-sectional study was carried out between August and October 2012 across six districts within the Dodoma Region in Tanzania, namely, Kondoa, Bahi, Dodoma Municipal, Chamwino, Kongwa and Mpwapa. Twenty sample HCF per district were identified through a mixture of simple and systematic random sampling. From all 270 public HCF in the six districts of Dodoma, 120 facilities were randomly included.

### Data collection and sample size

Data for this study were collected either prospectively, on the day of the visit to the facility, or retrospectively, from facility records dating back up to 1 year. Prospective data were collected by reviewing patients’ notebooks (which serve as both patient files and prescriptions) when they were handed in for dispensing after consultation. In addition to observing the standard recording of the patient’s name, age, sex, location, date, prescriber’s signature, treatment and diagnosis, any evidence of history taking, physical examination and laboratory investigation was also recorded. Recording the diagnosis was a prerequisite in order to assess whether the treatment followed the national STG. The same information was collected from randomly selected retrospective data in patient ledgers over the one-year period. However, in this case, it was not possible to get evidence on history, physical examination or laboratory investigations.

The intended sample size for prospective and retrospective data was 30 per facility. Three groups were classified according to the level of adherence to Tanzania’s 2007 STG: i) complete adherence to STG; ii) partial adherence; and iii) non adherence. An analgesic prescribed as additional medication was not considered wrong (in this work wrong medicines were defined as those prescribed that do not conform to the STG for the relevant illness). Further, adherence to the STG was defined by comparing a given diagnosis with the indicated medicine(s) as per the STG. Doses, dosing intervals and duration of treatments were not explored. A total of 2886 patient cases were recorded and analysed. The most frequently diagnosed illnesses, as documented in patient notebooks and patient records, were summarized in 25 broadly defined main illness groups that were coded accordingly (see supplementary information). However, only a selection of the nine most prevalent and locally relevant diagnoses were analysed in detail. The acute respiratory infection (ARI) category was subdivided into pneumonia and upper respiratory tract infections (URTI). Bronchitis was assigned to URTIs. Adherence to national STG was assessed for these nine groups. Primary diagnoses (the main concern and diagnosis for a patient’s visit) were analysed in detail. Further secondary and tertiary diagnoses, for lesser accompanying ailments, were not compared for the purposes of this paper.

Twelve pharmacy graduates from St John’s University in Dodoma were trained to carry out the study in a three-day training session which included pilot testing and revising the tools. Regular monitoring sessions assured quality standards in data collection as per instructions. The assessment on adherence to STG was reviewed and guided by a senior pharmacist experienced in similar assessments.

### Data processing and analysis

The manually filled and completed study tools and questionnaires were collected and double-entered into an Access 2010 database then transformed, checked, cleaned and summarized in Epi Info™ 7 and Stata/IC 12.1.

### Ethical considerations

The ethical clearance for the study was given by St John’s University of Tanzania, Directorate of research and consultancy, internal review committee on 16 October 2012.

## Results

### Overview of study sample and recorded data

In total, 2886 patient cases were recorded by data collectors, 784 (27.2%) prospectively, 1609 (55.8%) retrospectively and for 493 cases (17.1%) this information was missing. Of the 2886 patient cases recorded, 2554 included information on both diagnosis and treatment. The remaining 332 cases consisted of 318 with information on treatment but no diagnosis and 14 cases without either information.

Just under a half of all patients recorded (*n* = 1377; 47.7%) were children, 1231 (42.7%) were adults and for 278 (9.6%) data in this category were missing. The proportion of records per district was also calculated with Kondoa producing 551 (19.1%) study records, Bahi 377 (13.1%), Dodoma Municipal 252 (8.7%), Chamwino 553 (19.2%), Mpwapwa 553 (19.2%) and Kongwa 600 (20.8%). According to data collected from clinicians’ records, females appeared to represent the majority of primary diagnoses with 1202 (41.7%) cases, while 927 (32.1%) were males. For an additional 631 cases (21.9%) sex was not recorded by the clinician and for the remaining 126 (4.4%) these data were missing from the data collectors’ questionnaires (Table 1). In addition, 399 (13.8%) patients underwent a physical examination and 288 (10.0%) patients underwent a laboratory investigation (Table 1). These were mostly malaria rapid diagnostic tests or urine and stool examinations.

**Table 1:** Overview of information recorded by clinician (*N* = 2886)

	Recorded by clinician	Not recorded by clinician	Unknown^a^
Age	2556 (88.6)	281 (9.7%)	49 (1.7%)
Sex	2129 (73.8%)	631 (21.9%)	126 (4.4%)
Location	2522 (87.4%)	320 (11.1%)	44 (1.5%)
Date	2819 (97.7%)	32 (1.1%)	35 (1.2%)
History	822 (28.5%)	1975 (68.4%)	89 (3.1%)
Physical Examination	399 (13.8%)	2353 (81.5%)	134 (4.6%)
Lab investigation	288 (10.0%)	2475 (85.8%)	123 (4.3%)

^a^Data missing from data collection sheets

### Distribution of diagnoses

**Fig. 1:**
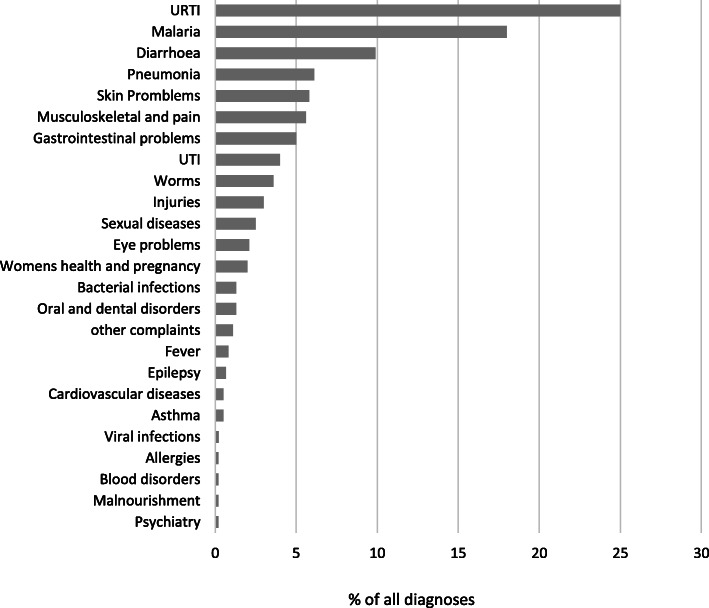
Distribution of all diagnoses over 25 illness groups and their relative percentage. Legend: *URTI* Upper respiratory tract infections; *UTI* Urinary tract infections

The 2886 cases recorded by the clinicians and data collectors were classified into one of the 25 illness groups (Fig. 1 and supplementary information). Of the 2554 diagnoses specified by the clinicians 2502 (86.7%) were primary diagnoses and 52 (1.8%) were, for unknown reasons, not classified.

Nine conditions were selected for deeper analysis based on prevalence and local significance such as eye diseases, a very common diagnosis in Dodoma region due to arid weather conditions. Musculoskeletal diagnoses and injuries which included a variety of conditions poorly addressed in the STG were excluded from this in depth analysis. Similarly, sexually transmitted infections were also excluded as they were addressed by a special programme for syndromic case management. Therefore, the nine selected diseases analysed in detail were, in order of prevalence, URTI, malaria, diarrhoea, pneumonia, skin problems, gastrointestinal problems (GI), urinary tract infections (UTI), worms and eye problems.

Comparing the number of all nine diagnoses using a Chi2 test, females were diagnosed significantly more than males (*p* < 0.001). There was no significant difference (*p* = 0.6) testing GI problems and UTI separately. However, a trend was observed with females suffering more often from GI problems compared with men (65% vs 35% respectively) and from UTI (71% vs 29%). When comparing adults and children’s primary diagnoses, GI problems were more prevalent in adults (68%) than in children (25%); 7% of the subjects’ ages were unknown. The percentage of primary diagnoses of UTIs in adults was 59% compared with 31% of children and 9.7% of the subjects’ ages were unknown. Similar proportions applied to the other seven disease groups, generally demonstrating a distribution of ca. two thirds of the primary diagnoses in children vs one third in adults with up to a 10% proportion of ages unknown.

**Fig. 2:**
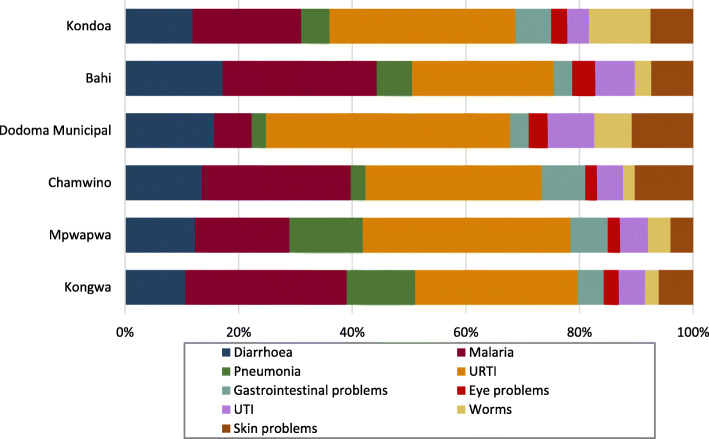
Distribution of primary diagnoses among the six districts. Legend: *URTI* Upper respiratory tract infections; *UTI* Urinary tract infections

Primary diagnoses were distributed among the six districts (Fig. 2). In five out of six districts URTIs were the most frequent diagnosis, only in Bahi was malaria diagnosed marginally more.

### Adherence to national standard treatment guidelines

**Fig. 3:**
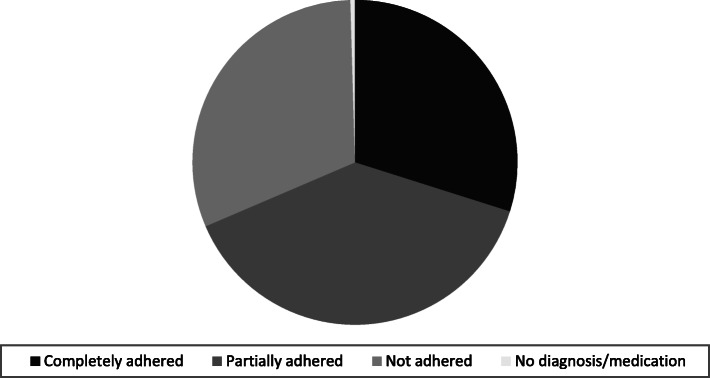
Adherence to standard treatment guidelines for primary diagnosis of 25 illness groups

For the 2502 primary diagnoses, information on adherence was available for 2004 cases. Complete adherence to STG for all 25 illness groups was recorded in 599 (29.9%) cases. Partial adherence was observed in just over a third (775 or 38.7%) of cases where patients received the correct medication but additional unnecessary or wrong medicines. Non-adherence to STG was found in 621 (30.9%) cases. In nine (0.5%) cases no diagnosis/medication was given (Fig. 3).

**Fig. 4:**
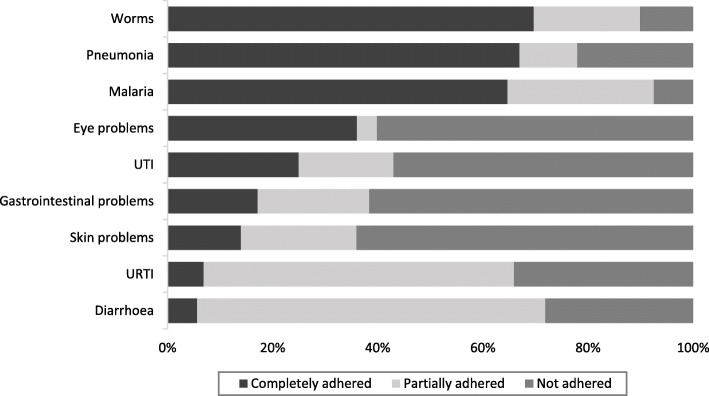
Adherence to standard treatment guidelines for primary diagnosis. Legend: *URTI* Upper respiratory tract infections; *UTI* Urinary tract infections

Complete adherence of primary diagnoses to STG for the nine selected conditions was highest when worms were diagnosed and lowest for diarrhoea. The proportion of cases that were incorrectly treated was highest in patients with skin problems and lowest for malaria (Fig. 4).

**Fig. 5:**
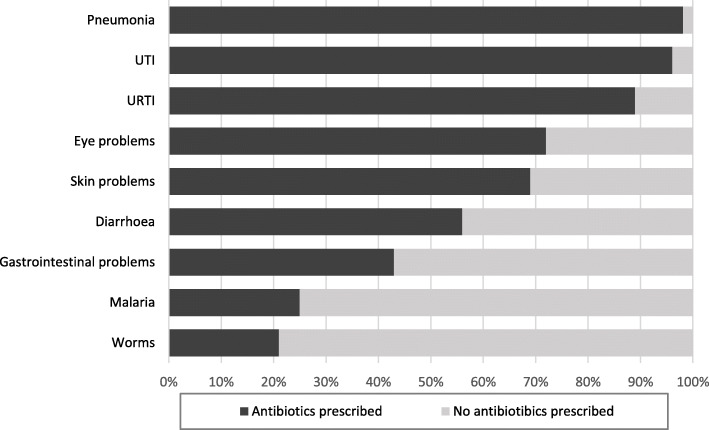
Prescribing of antibiotics. Legend: *URTI* Upper respiratory tract infections; *UTI* Urinary tract infections

The adherence to STG for age and sex were stratified and no important differences could be found. High prescribing of antibiotics was observed with 61.2% of all patients having an antibiotic prescribed either as a sole treatment or as an additional treatment (Fig. 5).

### Diagnosis and distribution for non-communicable diseases

Only 51 (2%) non-communicable diseases (NCDs) were recorded among the 2554 diagnoses. Adherence to STG was high in three categories: epilepsy, asthma and psychiatric disorders; however, not a single case of cardiovascular disease was treated correctly. Epilepsy was diagnosed in 17 cases. In 13 of those cases the STG were completely adhered to. There were no instances of partial adherence; however, three cases did not adhere at all and in one case the information was missing. Cardiovascular diseases were diagnosed in 13 cases. None of the diagnoses completely adhered to the STG, six partially adhered but seven did not at all. Asthma was recorded in 13 cases. In 10 of those cases there was complete adherence, there was partial adherence in only one case and non-adherence in another two cases. Complete adherence to STG was logged in three out of the five psychiatric disorders diagnosed, one case of non-adherence was noted and in one case the information was missing. In the only case of diabetes that was diagnosed the STG were not adhered to.

## Discussion

The three most prevalent diagnoses in this study were ARI, malaria and diarrhoea. URTI and diarrhoea diagnoses and treatments adhered least to STG. These findings coincide with studies that seem to suggest that low adherence to STG, especially for the management of childhood diseases such as diarrhoea and respiratory tract infections, is common not only in LMICs but worldwide [20–22]. Partial adherence to STG for URTI was mainly due to the high prescription of antibiotics for bronchitis which is generally caused by a virus and would therefore not benefit from them. In accordance with STG, antibiotics were indicated for the treatment of pneumonia. They were not, however, indicated for URTIs and GI problems. Fifty-six percent of diarrhoea cases, 25% of malaria cases and 21% of worm cases also received an antibiotic. However, in rural areas diagnosis is complicated by the scarcity of diagnostic tools and facilities, which can lead to prescribing antibiotics presumptively when rapid decisions are a matter of mortality. This was observed, at least, for malaria treatment in rural areas, where patients presenting with fever were more likely to receive co-prescription of antibiotics and antimalarial when diagnosis was clinical and not backed by malaria rapid testing [23]. Nevertheless, the fact that 61% of patients in this study received an antibiotic, disregarding the diagnoses, is of grave concern. It is also a far cry from the WHO prescribing indicator that recommends their use in less than 30% of cases [2, 24]. Tanzania has a history of overprescribing antibiotics and according to an assessment conducted in four regions of Tanzania in 2014 it does not appear to have improved [3]. AMS programmes that have positively affected the behaviour of clinicians’ antibiotic utilization and proven to be effective in high-income countries have also been introduced to African countries with some success [16, 25–27]. One such programme was initiated in Mbeya Zonal Referral Hospital of Tanzania [28, 29]. A baseline assessment of antibiotic prescription in this training hospital revealed that prescribers adhered to the Tanzania STG recommendations for antibiotic choice 63% of the time, but it decreased to 15% when treatment duration was taken into account. Successful AMS relies on a commitment from facility management leadership and accountability. AMS programmes usually comprise a multidisciplinary committee ensuring prudent antibiotic use [16, 30, 31]. To implement an AMS programme in PHC facilities, however, will be more of a challenge in Tanzania considering the shortage of staff, insufficient guidance and supervision and frequent stock-outs of medicines.

The total of all malaria diagnoses in all districts in this study was 18%. It is interesting to note the lower prevalence of malaria, 1.8% of all diagnoses, in the Dodoma Municipal which is a more urban district. Complete adherence to STG for malaria for primary diagnoses was 65%, partial 28% and non-adherence 7.5%. Budimu et al. reported, from a study conducted in the Meatu district of Tanzania in 2017, 54.6% of all 196 healthcare workers there strictly adhered to the STG for malaria case management. Ten (5.1%) healthcare workers partially adhered when they chose antimalarials without confirmed cases of malaria and 79 (40.3%) health workers did not adhere [15]. Although the study in the Meatu district was on a much smaller scale and in a different region, comparing these studies, the adherence to STG seems to have regressed. In a study in the Kilosa district of Tanzania back in 2010, concerns were raised that the STG for administering the malaria treatment, artemisinin combination therapy, were not clear enough and thereby probably contributed to the prescribers’ non-conformity with STG [32].

Complete adherence to diarrhoea STG was extremely low. Partial adherence was mainly due to prescribing oral rehydration solution (ORS) plus antibiotics and the lack of prescribed Zinc. Extremely low adherence to STG for the management of acute diarrhoea in children under 12 was also found in a study in Ujjain, Madhya Pradesh, India and the high rate of prescribing non-recommended medication was discussed [20]. The duration and volume of diarrhoea is not lessened with ORS, therefore, many practitioners look for alternative therapies to shorten its time span [33]. As vomiting can be caused and aggravated by incorrectly prepared ORS, parents and caregivers may be discouraged to continue the therapy leading to a failure in oral hydration [34]. Thus, the perceived ineffectiveness of ORS therapy may have then led to an increase in prescribing other non-recommended medications such as antibiotics. Pathak et al. also considered that accompanying symptoms like the presence of fever, pain, blood in the stool and vomiting significantly increased antibiotic prescribing even though most diarrhoeal episodes are of a viral origin [20].

Another matter of interest in the present study was the low number of diagnoses of NCDs with just 51 cases (2%) despite NCDs such as heart disease, stroke, cancer, chronic respiratory diseases and diabetes being the leading cause of mortality in the world today. In fact, recent data shows that NCDs are estimated to account for 33% of all deaths in Tanzania [35]. Thus, the low number of diagnoses in the present study may be explained with either actual low prevalence in the Dodoma region at the time or, what is more likely, low awareness and insufficient diagnostic skills of what are often, initially, silent diseases. Similarly, mental disease appears not to have been a problem in Dodoma region. This again may be due to low prevalence but more likely to be an unawareness and underdiagnoses of mental conditions.

The implementation of STG provides a point of reference by which practitioners can review, compare and advance the quality of care that they deliver. They are packaged so as to contain statements that provide expected standards of practice in order to diminish variations in clinical practice and to reduce costly and avoidable mistakes and adverse events [36]. It is of concern then that, approximately, only a third of primary diagnoses in this study were prescribed and treated completely in accordance with the national STG. A little over a third (38.7%) of primary diagnoses prescriptions partially adhered to them, thus, in these cases patients at least received the correct medicine but also further unnecessary or incorrect medicines, which is a waste of limited resources. In addition, approximately a third of prescribers diagnosed and treated patients incorrectly and not in accordance with STG; therefore the quality of care and patient outcome may have been seriously compromised. Deviation from clinical guidelines may be due to various factors such as conscious clinical decision-making, empirical prescribing habits, resource availability limitations caused by supply challenges and lack of familiarity or availability of guidelines.

As printed materials alone seem to have little effect in changing the prescribing behaviour of clinical health workers, STG need to be accompanied by reminders, educational outreach and feedback in order to be effective [6, 11–13]. Notwithstanding, not implementing effective training and supportive supervision, the shortage of healthcare workers, together with high clinical and administrative workloads negatively impacts the quality of patient care delivered. In Tanzania, between the period 2007 and 2013 the physician to population ratio per 10,000 was 0.3. This was far lower than the WHO African Region average of 2.7 and the global average of 13.9. During the same period the nursing and midwifery personnel to population ratio per 10,000 was 4.4. Again, this was far lower than the WHO African region average of 12.4 per 10,000 and the global average of 28.6 [37]. The low ratio of healthcare professionals, together with an expanding population, shortage of medical commodities and increasing health burdens from chronic and emerging diseases, cause undue pressure on healthcare staff. Health personnel, already operating at (or beyond) their limit, will find it even harder to find the time for comprehending a complex 450-page document such as Tanzania’s STG, which further contributes to the wider lack of reference and adherence [38].

### Study limitations

Diagnoses were accepted as written by clinicians and were not assessed for correctness. Thus, it is assumed that the diagnoses were correct. In many cases symptoms rather than diagnoses were noted, as for instance pain or fever. Therefore it was impossible to assess the underlying illness and corresponding suitability of therapy. In two cases of fever and pain, correct treatment according to STG was assumed to be an analgesic or antipyretic respectively regardless of underlying pathology. Moreover, as some patients had multiple diagnoses, wrong medications could not be clearly assigned to a specific diagnosis. They were usually assumed to belong to the secondary or tertiary diagnoses.

In some facilities, the number of patients was very small and the target number of 30 could not be reached. Also, due to communication limitations, three facilities in Kondoa district and one facility in Mpwapwa district had to be replaced on the day of visit. The nearest dispensaries were visited instead.

Finally, there are limitations regarding the evidence collected in the framework of this study. First, this is a descriptive report and only such conclusions can therefore be drawn; second, the study was conducted in 2012 and some data is missing, therefore, the article presents data that may not accurately reflect the current situation. Nevertheless, other subsequent studies and the authors’ continued experience in Tanzania indicates that the situation has not significantly changed. Moreover, the current study fills an important gap owing to the scarcity of relevant studies in this area in Tanzania. To the best of our knowledge this is still the only study that covers adherence to STG in Dodoma region in such detail.

## Conclusion

Prescribers’ general adherence to national STG in PHC facilities in the public sector in Dodoma Region was found to be very low. Clinical evidence-based guidelines such as STG are of little value if not implemented and adhered to. Tanzania’s underfunded health system does not provide for printing, delivering and mentoring of new STG editions for all prescribers in all health facilities in Tanzania, especially in remote rural areas. Furthermore, PHC facilities are strained from an excess of patients from an expanding population. The complex STG, often written for specialists in secondary or tertiary level care, are difficult to interpret and implement for lower cadre prescribers in the peripheral healthcare level. These are among many concerns likely to have an effect on prescribers’ noncompliance with the clinical guidance STG can provide. Poor prescribing practice diminishes quality of care and increases the chances of poor health outcomes. Multicomponent interventions, similar to the AMS programmes for antibiotics, incorporating prescriber training and patient education, complemented with supportive supervision should be implemented. Greater commitments from the government and stakeholders are consequently required to strengthen the health system and expand the financial and human resources available.

## Supplementary Information

**Additional file 1: Supplement Table.** Illness group and Diagnoses as recorded by prescriber/clinician.

## Data Availability

The data sets used and/or analysed during the current study are available from the corresponding author on reasonable request.

## Abbreviations

UHC: Universal healthcare
SDG: Sustainable development goal
STG: Standard treatment guidelines
LMICs: Low- and middle-income countries
PHC: Primary healthcare
HCF: Healthcare facilities
HPSS: Health Promotion and System Strengthening Project
ARI: Acute respiratory infection
URTI: Upper respiratory tract infections
GI: Gastrointestinal
UTI: Urinary tract infections
NCDs: Non-communicable diseases
ORS: Oral rehydration solution
AMS: Antimicrobial stewardship
