# Supplementary material for: Adherence to standard treatment guidelines among prescribers in primary healthcare facilities in the Dodoma region of Tanzania
Source: BMC Health Serv Res. 2021 Mar 24;21:272. doi: 10.1186/s12913-021-06257-y (PMC7990379; doi:10.1186/s12913-021-06257-y)
Supplement: Supplementary file 1 — Additional file 1: Supplement Table. Illness group and Diagnoses as recorded by prescriber/clinician. [file 12913_2021_6257_MOESM1_ESM.docx]

| **Illness group** | **Diagnoses as recorded by prescriber/clinician** |
| --- | --- |
| Diarrhoea | Diarrhoea, Dysentery, Gastroenteritis, Enteric Fever |
| Malaria | Malaria |
| Pneumonia | Pneumonia- LRTI |
| Upper respiratory tract infections (URTI) | Bronchitis, cough, coryza, rhinitis, otitis media, flu, cold, sinusitis, tonsillitis, ear infection |
| Gastrointestinal tract conditions (GIT) | Gastritis, abdominal pain, gastric pain, stomach pain, heart burn, dyspepsia, irritable bowel syndrome, indigestion, peptic ulcer |
| Eye diseases | Conjunctivitis, eye infection, trachoma, red eyes, eye injury |
| Urinary tract infections (UTI) | UTI |
| Worms and parasites | Hookworm, worms, helminthiasis, schistosomiasis, giardiasis, taeniasis, amoebiasis |
| Skin diseases | Abscess, boils, scabies, dermatitis, fungal infection, dermatomycosis, herpes simplex, herpes, zoster, skin rash, impetigo, skin disease, skin infection, tinea capitis, tinea corporis |
| Musculoskeletal and pain | Accident, soft tissue injury, arthritis, bruise, fracture, back pain, lumbago, sprain, headache, fibrositis |
| Women’s health and pregnancy | Abortion, complication in pregnancy, infertility, amenorrhea, dysmenorrhea, mastitis |
| Sexual diseases | Vaginal discharge syndrome (VDS), syphilis, Sexually transmitted disease (STD), Genital discharge syndrome (GDS) |
| Oral and dental disorders | Oral infection, toothache |
| Psychiatric disorders | Psychiatric case, depression |
| Malnourishment | Vitamin deficiency, pellagra |
| Blood disorders | Anaemia |
| Injuries and wounds | Cut wound, trauma, dog bite, snake bite, burn |
| Allergies | Allergy |
| Epilepsy | Epilepsy |
| Asthma | Asthma |
| Fever | Fever |
| Viral infections | Mumps, chicken pox, small pox |
| Bacterial infections | Sepsis, bacterial infection, septic wound, local infection, anthrax |
| Cardiovascular diseases | Chest pain, angina, hypertension |
| Diabetes | Diabetes |
| Other complaints | All other diagnoses |
